# Drug Decriminalization, Fentanyl, and Fatal Overdoses in Oregon

**DOI:** 10.1001/jamanetworkopen.2024.31612

**Published:** 2024-09-05

**Authors:** Michael J. Zoorob, Ju Nyeong Park, Alex H. Kral, Barrot H. Lambdin, Brandon del Pozo

**Affiliations:** 1 Rhode Island Hospital, Providence; 2 Warren Alpert Medical School of Brown University, Providence, Rhode Island; 3 RTI International, Berkeley, California

## Abstract

**Importance:**

With the implementation of Measure 110 (M110) in 2021, Oregon became the first US state to decriminalize small amounts of any drug for personal use. To date, no analysis of the association of this law with overdose mortality has fully accounted for the introduction of fentanyl—a substance that is known to drive fatal overdose—to Oregon’s unregulated drug market.

**Objective:**

To evaluate whether the decriminalization of drug possession in Oregon was associated with changes in fatal drug overdose rates after accounting for the rapid spread of fentanyl in Oregon’s unregulated drug market.

**Design, Setting, and Participants:**

In this cohort study, the association between fatal overdose and enactment of M110 was analyzed using a matrix completion synthetic control method. The control group consisted of the 48 US states and Washington, DC, all of which did not decriminalize drugs. The rapid spread of fentanyl in unregulated drug markets was determined using the state-level percentage of all samples reported to the National Forensic Laboratory Information System that were identified as fentanyl or its analogues. Mortality data were obtained from the Centers for Disease Control and Prevention for January 1, 2008, to December 31, 2022. Data analysis was performed from fall 2023 through spring 2024.

**Exposures:**

Measure 110 took effect in Oregon on February 1, 2021.

**Main Outcomes and Measures:**

The primary outcome assessed was fatal drug overdose rates per half-year. A changepoint analysis also determined when each state experienced a rapid escalation of fentanyl in its unregulated drug market.

**Results:**

In this analysis, rapid spread of fentanyl in Oregon’s unregulated drug supply occurred in the first half of 2021, contemporaneous with enactment of M110. A positive crude association was found between drug decriminalization and fatal overdose rate per 100 000 per half year (estimate [SE], 1.83 [0.47]; *P* < .001). After adjusting for the spread of fentanyl as a confounder, the effect size changed signs (estimate [SE], −0.51 [0.61]; *P* = .41) and there was no longer an association between decriminalization and overdose mortality in Oregon. Sensitivity analyses were consistent with this result.

**Conclusions and Relevance:**

In this cohort study of fatal drug overdose and the spread of fentanyl through Oregon’s unregulated drug market, no association between M110 and fatal overdose rates was observed. Future evaluations of the health effects of drug policies should account for changes in the composition of unregulated drug markets.

## Key Points

**Question:**

Is the 2021 Oregon law that decriminalized drug possession associated with overdose mortality when accounting for the spread of fentanyl through the state’s unregulated drug market?

**Findings:**

In this cohort study of fatal overdose and fentanyl spread through Oregon’s unregulated drug market, decriminalization of drug possession was not associated with an increase in fatal drug overdose rates in Oregon in the 2 years after its enactment.

**Meaning:**

The findings of this study suggest that when evaluating the association of public policies with overdose mortality, it is critical to account for the role of fentanyl as the principal driver of the overdose mortality epidemic in the US.

## Teaser

This cohort study examines the association of decriminalization of drug possession with fatal drug overdose rates in Oregon while accounting for the rapid spread of fentanyl in the state’s unregulated drug market.

## Introduction

Amid the worst fatal opioid overdose mortality crisis in US history,^1^ many jurisdictions have explored alternatives to traditional models of arrest and incarceration for drug possession in acknowledgment of the health harms that can result from opioid overdose.^2,3,4^ At the street level, states have decriminalized the personal possession of selected federally scheduled substances (eg, cannabis, psilocybin, and buprenorphine), enacted Good Samaritan laws to shield people from arrest at the scene of an overdose when they seek help,^5,6^ created police programs that link people to treatment and harm reduction services,^7,8^ and taken de facto approaches to decriminalizing drug possession.^9,10^ In November 2020, in the most sweeping response to date, Oregon voters passed Measure 110 (M110), making it the first state to de jure decriminalize the possession of all nonprescribed drugs for personal use, while reallocating millions of dollars toward addiction treatment, recovery programs, housing, and harm reduction services.^11^ The measure intended to reduce overdose by expanding and promoting linkages to health care systems for people who use drugs, reducing entrenched racial and ethnic disparities in the enforcement of drug possession laws in doing so.^12^

Since its enactment, however, M110 faced implementation challenges,^13^ and addiction treatment capacity did not sufficiently expand to meet the state’s needs.^14^ Police officials reported that decriminalization hampered their ability to address concerns about public drug use,^15^ and in 2021, Oregon’s rate of fatal overdose increased by approximately 50% compared to the previous year.^16^ In response to these initial outcomes, Oregon’s legislature recriminalized drug possession in March 2024, to take effect in September 2024.^17^ As other jurisdictions look to Oregon’s health outcomes in considering their own policy responses to overdose going forward, an accurate assessment of the association between M110 and fatal overdose is critical.

To date, 2 studies^18,19^ have assessed the effects of M110 on overdose mortality. Spencer concluded that M110 “caused 182 additional unintentional drug overdose deaths to occur in Oregon in 2021,”^18^^(p1)^ representing a 23% increase. Joshi et al^19^ used a model with restricted Centers for Disease Control and Prevention (CDC) mortality data for both intentional and unintentional deaths, and they did not detect a change attributable to decriminalization. However, neither study fully accounted for the third wave of the overdose mortality crisis reaching Oregon: the supply-side shock of illicitly manufactured fentanyl, a highly potent synthetic opioid.^20^ The former study did not mention fentanyl at all, and the latter used total fentanyl seizure counts from 2018 to 2019 only to test the fit of its model. Yet the rapid spread of fentanyl in unregulated drug markets dramatically increases overdose mortality,^21,22^ marking a “significant shift in the structural risk environment” for people who use illicit opioids.^23^^(p108)^ As fentanyl spread throughout the nation, the overdose fatality rate involving synthetic opioids other than methadone increased from 1.8 per 100 000 in 2014 to 21.8 in 2021, a 1200% increase.^24^ However, fentanyl did not saturate each state’s unregulated drug market at the same time^25^; from about 2013 onward, it spread from east to west over the course of several years.^20,26^ Therefore, to address its potential confounding effects, studies that evaluate associations between a given intervention and overdose should account for the heterogenous spread of fentanyl across the US. There has yet to be a study that does so for M110.

In an effort to fill that gap, this study assessed changes in fatal overdose rates in Oregon after the implementation of M110 while accounting for the timing of the rapid spread of fentanyl through the state’s unregulated drug market. To provide context, an additional analysis was conducted to assess trends in fatal overdose during the contemporaneous period of decriminalization and recriminalization of drug possession in the neighboring state of Washington. These analyses intended to provide researchers, public health officials, and policymakers with an assessment of the association of fentanyl with fatal overdose in the US Pacific Northwest during its period of drug decriminalization and, in the case of Washington, recriminalization.

## Methods

Because this cohort study used publicly available data released in the aggregate, it did not constitute human participant research and was exempt from institutional review board review per the Common Rule. The study followed the Strengthening the Reporting of Observational Studies in Epidemiology (STROBE) reporting guideline as appropriate for a difference-in-differences design.

### Approach

This study used the matrix completion method^27^ to impute the counterfactual trend in fatal overdose (ie, fatal overdose rates in Oregon had M110 not occurred) while accounting for the state’s rapid spread of fentanyl in its unregulated drug market as a time-varying covariate. The matrix completion estimator—a causal inference technique that emerged from machine learning literature—creates a lower-rank approximation of the outcome data matrix using information from untreated observations (and, importantly, allows for conditioning on time-varying factors).^27^ The study modeled what Oregon’s overdose rate would have looked like in the postdecriminalization period had decriminalization not taken place; it did so by using trends in overdose mortality rates in Oregon and other states as well as the observed association between rapid changes in fentanyl seizures and mortality across all states.

### Data Sources

This study leveraged 2 sources of publicly available administrative data. To measure overdose mortality, we utilized unrestricted multiple cause of death mortality data from the CDC Wonder database aggregated to the half-year (ie, 6-month) level between January 1, 2008, and December 31, 2022, the most granular interval for which the drug seizure data described in the next paragraph is available. This represents 2 years of postintervention data, 1 additional year beyond the analysis period of Spencer^18^ and 9 months beyond that of Joshi.^19^ Our primary dependent variable consisted of all fatal drug poisonings (ie, accidents, suicides, homicides, and unknown intent); the corresponding cause of death classifications are listed in the eMethods in Supplement 1. Direct replications of Spencer^18^ used monthly level underlying cause of death mortality data restricted to unintentional drug poisonings from January 1, 2018, to December 31, 2021.

The National Forensic Laboratory Information System (NFLIS) is a federal repository of law enforcement drug identification incident records provided by forensic laboratories in all 50 states and Washington, DC. To construct a state-level proxy for fentanyl in illicit drug supplies, we utilized data from the NFLIS Public Query System. Specifically, we queried all fentanyl-related substances for all states for half years (the most granular temporal unit available in the tool) from 2008 to 2022.

### Measures

We used the share of law enforcement submissions to the NFLIS that were for fentanyl or a related substance as a state-period level proxy measure of illicit fentanyl in drug supplies. We calculated the state-level percentage of all drug seizures that were for fentanyl or related substances for each half-year period between 2008 and 2022. We used this percentage rather than the overall volume of submissions to limit the effects of secular differences in state-level narcotics enforcement; robustness tests (eMethods in Supplement 1) explored alternative metrics of fentanyl exposure. To assess this proxy’s association with fatal overdose, we estimated the association between state overdose mortality rates and our calculated NFLIS fentanyl percentages.

Because M110 took effect on February 1, 2021, we conceptualized the treatment period as commencing in the first half of 2021 or, in monthly analyses, as commencing in February 2021. Following previous studies,^18,19^ we did not include Washington State in the comparison group because its supreme court effectively decriminalized drug possession for 4 months between February 25 (when it struck down the state’s drug possession law) and July 25, 2021, when misdemeanor recriminalization took effect. Rather, as an ancillary analysis, we present the resulting associations among decriminalization, recriminalization, and fatal overdose in Washington State using a synthetic control model, and we also replicate Spencer’s^18^ separate analysis of the state.

### Statistical Analysis

#### Changepoint Analysis

To assess whether a supply-side fentanyl shock in the unregulated drug market was associated with the exposure and outcome variables, we used a changepoint detection procedure to identify the point in time (if any) for each state when there was the most profound change in the mean of the distribution of the percentage of fentanyl reports to the NFLIS. This procedure was implemented with the changepoint package in R, version 4.2.0 (R Project for Statistical Computing),^28^ with the default “at most one change” setting and the default modified bayesian information criterion penalty. We plotted states according to their centroid degree longitude and the changepoint date of a mean shift in the percentage of fentanyl in drug seizures to visualize the geographic spread of fentanyl and the extent to which it coincided with Oregon’s decriminalization.

#### Two-Way Fixed-Effects Regression

To determine whether the timing of the rapid spread of fentanyl in the unregulated drug market was associated with overdose mortality, we used state-level half-year panel data for all 50 states and Washington, DC, to estimate the association of fentanyl exposure with overdose mortality. We estimated 1-way (state-only; period-only) and 2-way (state and period) fixed-effects regression models and, in the final model, included state-specific linear time trends. In all cases for our study, significance was prespecified as 2-sided, with *P* < .05 as the threshold.

#### Matrix Completion Synthetic Control Method

Next, we analyzed the change in overdose mortality in Oregon relative to a synthetic control. We used the cross-validation procedure in the R package fect to select the estimator that minimizes the mean squared prediction error. The cross-validation procedure selected the matrix completion estimator, which leverages the pretreatment control unit outcomes and covariates to estimate the unobserved counterfactual outcome of the treated unit under no treatment in the posttreatment period. Because the number of treated units was small, we used jackknife SEs clustered at the unit (ie, US state) level.

#### Supplementary Analysis

As sensitivity checks, we estimated the association between decriminalization and overdose mortality (first unadjusted and then adjusted for fentanyl exposure) using alternative causal panel data models that incorporated time-varying covariates: the 2-way fixed-effects model, the generalized synthetic control method,^29^ and interactive fixed-effects methods.^29^ We then replicated the difference-in-differences (2-way fixed-effects regression) results in Spencer 2023,^18^ which reported decriminalization increased overdose mortality in Oregon, and extended these analyses by incorporating state-level fentanyl exposure as a covariate.

Finally, to examine the ancillary hypothesis that recriminalizing drug possession after a period of decriminalization might slow or reverse ongoing increases in a state’s rate of fatal overdose, we used the aforementioned matrix completion synthetic control method to analyze trends in Washington State during its decriminalization period and after the state legislature recriminalized drug possession as a misdemeanor offense in July 2021.

We used R statistical software, version 4.2.0 (R Project for Statistical Computing), for all analyses. Data analysis was performed from fall 2023 through spring 2024.

## Results

### Changepoint Analysis of the Rapid Spread of Illicit Fentanyl in the Unregulated Drug Market

**Figure 1.**
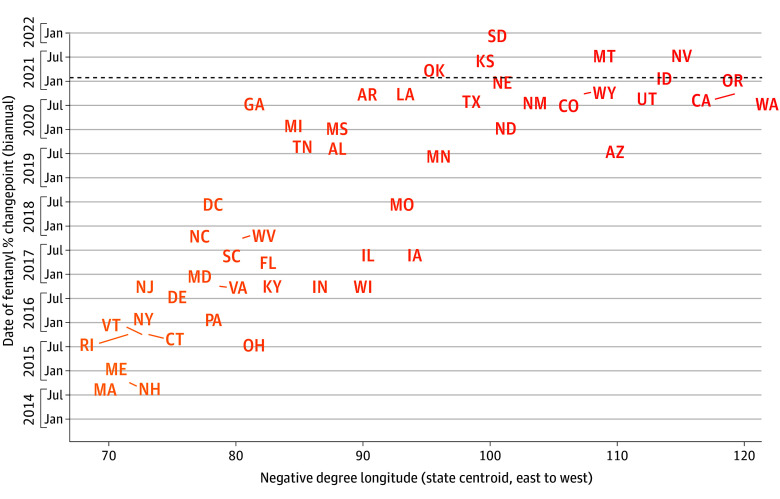
Geographic Spread of Fentanyl Across US State-Level Unregulated Drug Markets, 2014 to 2022 Dates of the rapid spread mean shift of fentanyl in the unregulated drug market are presented by state (with states represented by their standard postal abbreviations) per half year (biannual). The horizontal line indicates the date that drug decriminalization took effect in Oregon. States are arranged by the (negative) longitude of their geographic centroid, meaning that a rightward shift in the graph corresponds to western distance. The R package changepoint^28^ was used to detect the dates at which there was the most profound mean shift in the percentage of law enforcement records that contained fentanyl submitted to the National Forensic Laboratory Information System. Diagonal lines next to a state point to its most accurate position on the graph; some state locations were displaced by crowding.

Fentanyl appeared to spread rapidly in the unregulated opioid supply of the US between 2014 and 2021. Changepoints were earlier for eastern states than for western states (Figure 1). The earliest changepoints were in New England states in the second half of 2014. In the Pacific Northwest, the changepoint in Washington State occurred in the second half of 2020, and in Oregon in the first half of 2021, both after nearly all other states. Additionally, eFigure 1 in Supplement 1 shows each state’s trend in the percentage of drug seizures containing fentanyl, illustrating the same regional patterns.

The dashed horizontal line in Figure 1 indicates the date M110 took effect. The intersection in the figure of the time at which Oregon’s unregulated opioid market experienced a fentanyl supply shock and enactment of M110 indicates that the 2 events occurred contemporaneously.

### State-Level Illicit Fentanyl Saturation and Fatal Drug Overdose

**Figure 2.**
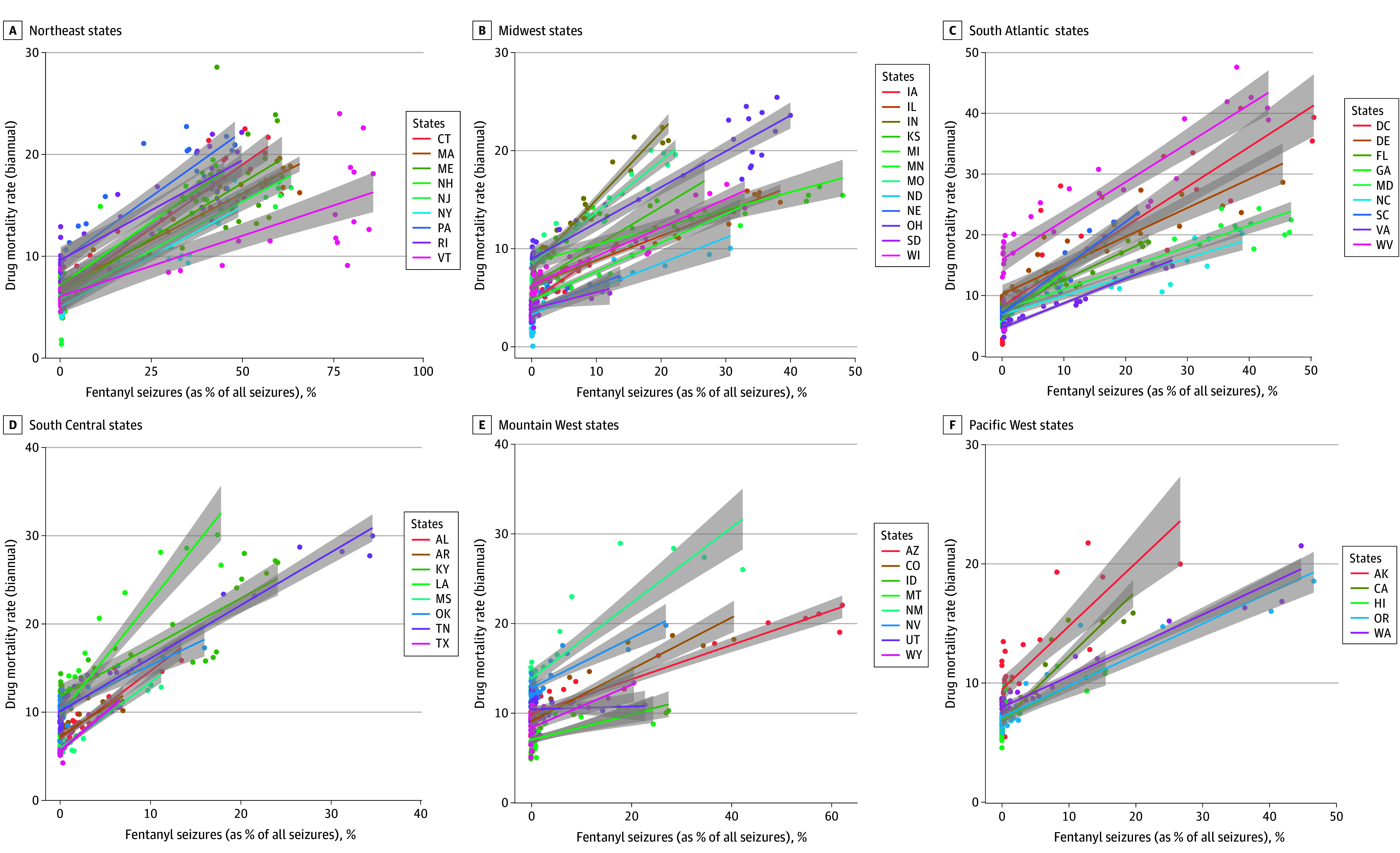
Within-State Associations Between Fentanyl Exposure and Overdose Mortality Rates by Region, 2008 to 2022 A to F, Association between the percentage of all National Forensic Laboratory Information System law enforcement seizures that contained fentanyl and drug overdose mortality rates, with states grouped by region: Northeast (A), Midwest (B), South Atlantic (C), South Central (D), Mountain West (E), and Pacific West (F). Each point represents a half-year (biannual) period in a state over the period from 2008 to 2022. In nearly all states, a positive association existed between fentanyl exposure and overdose mortality.

Analysis revealed a positive association between the percentage of NFLIS records containing fentanyl and overdose death rates across states, shown regionally in Figure 2 and for each state in eFigure 2 in Supplement 1. Table 1 demonstrates the consistency of this association across model specifications: the unadjusted bivariate association (model 1), adding unit-level (state) fixed effects (model 2), adding time-level fixed effects (model 3), and adding both state and time fixed effects (model 4). Model 5 adds a state-specific linear time trend. Across all models, the association between fentanyl saturation and overdose mortality was positive. Fentanyl seizures per person (eTable 1 in Supplement 1) and a combined index of the percentage of total fentanyl seizures and fentanyl seizures per person (eTable 2 in Supplement 1) were similarly associated with mortality.

**Table 1.** Fentanyl Saturation and Overdose Mortality Rates^a^

Dependent variable: overdose death rate	Model 1	Model 2	Model 3	Model 4	Model 5
Fentanyl, % (SE) of total seizures [95% CI]	0.25 (0.01) [0.23 to 0.26]^b^	0.27 (0.03) [0.21 to 0.33]^b^	0.17 (0.01) [0.15 to 0.18]^b^	0.16 (0.03) [0.10 to 0.22]^b^	0.12 (0.03) [0.06 to 0.17]^b^
Model characteristic					
State fixed effects	No	Yes	No	Yes	Yes
Time (half-year) fixed effects	No	No	Yes	Yes	Yes
State linear time trend	No	No	No	No	Yes
SE clustering	None	State	Half year	State	State
No. of observations	1530	1530	1530	1530	1530

^a^ Values are presented as regression coefficients with SEs and 95% CIs of fentanyl exposure (percentage of all NFLIS seizures) on overdose death mortality rates for various statistical model specifications (estimated with the R package fixest). The unit of analysis is state half year (eg, Alabama in H1 of 2010). The date range is 2008 H1 to 2022 H2. eTables 1 and 2 in Supplement 1 show similar results with alternative operationalizations of fentanyl saturation.

^b^ *P* < .05.

### Decriminalization, Fentanyl Saturation, and Overdose

**Figure 3.**
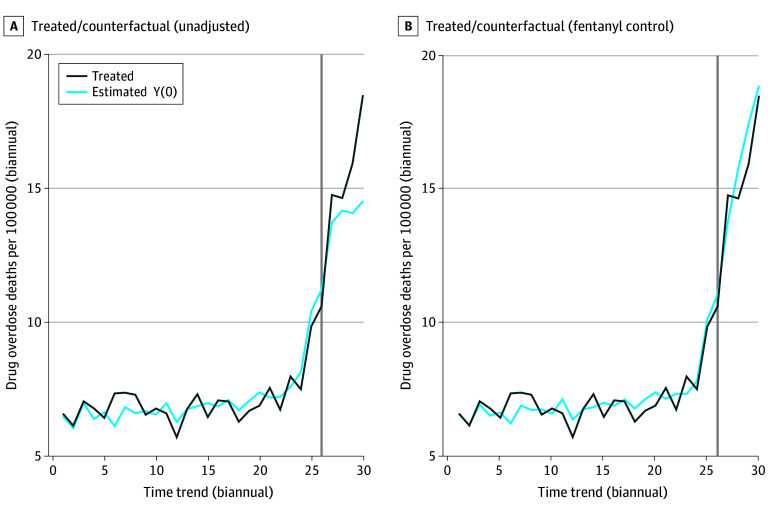
Matrix Completion Analyses for Oregon, 2008 to 2022 A and B, Analyses were unadjusted (A) and adjusted (B) for the rapid spread of fentanyl. In each plot, the blue line, labeled “Estimated Y(0),” represents the imputed counterfactual mortality rate for the estimated counterfactual Oregon according to the matrix completion model. The black line represents the observed overdose mortality rate for Oregon. The x-axis is the number of half years since 2008 (biannual; 1 indicates the first half of 2008; 30 indicates the second half of 2022). The y-axis shows the rate of drug-related deaths per 100 000 people per half year (the number of deaths in the 6-month period divided by the annual population, multiplied by 100 000). Plots were generated via the R package gsynth.

The actual and counterfactual overdose mortality rates in Oregon according to the matrix completion model are presented in Figure 3 and eTable 3 in Supplement 1. The plot in Figure 3A shows the unadjusted (ie, a model with no fentanyl control) association of decriminalization with overdose mortality rates in Oregon compared with a synthetic counterfactual, with the observed overdose rates substantially higher than the expected overdose rates (estimate [SE], 1.83 [0.47]; *P* < .001). However, incorporating state-level fentanyl exposure to account for confounding (Figure 3B) eliminated this result, with the counterfactual overdose rates hovering above the observed rates in the posttreatment period. Indeed, after adjusting for fentanyl, the average treatment effect on the treated over the posttreatment period changed signs (corresponding to lower mortality than expected), although the result was imprecise and not statistically significant (estimate [SE], −0.51 [0.61]; *P* = .41). A 95% CI of the decriminalization estimate ranged from −1.70 to 0.69 deaths per 100 000 people per half year, corresponding to a lower bound estimate of 289 fewer deaths in Oregon associated with decriminalization and an upper bound estimate of 117 excess deaths over the 2021 to 2022 period. In the model adjusting for fentanyl exposure, fentanyl case records reported to the NFLIS exhibited a positive association with overdose mortality (β [SE], 0.16 [0.03]; *P* < .001).

### Spencer’s Sensitivity to a Fentanyl Exposure Covariate

Table 2 and eFigure 3 in Supplement 1 present the results of regression models of decriminalization on overdose mortality with month and state fixed effects. The odd-numbered columns in Table 2 reproduce the overall results of Spencer,^18^ showing that statistical models unadjusted for fentanyl exposure produced positive associations between decriminalization and accidental overdose mortality. However, when we incorporated our fentanyl exposure variable in the models to assess the study’s sensitivity to this covariate, the estimated effect sizes of drug decriminalization were not significant in Oregon (estimate [SE], 0.09 [0.11]; *P* = .38), in Washington (−0.12 [0.16]; *P* = .44), and for both states combined (−0.007 [0.15]; *P* = .96). Fentanyl exposure was associated with increased overdose mortality in model 2 (estimate [SE], 0.02 [0.008]; *P* = .009) and in models 4 and 6 (estimate [SE], 0.02 [0.007]; *P* = .008).

**Table 2.** Difference-in-Differences Results Replication and Extension^a^

Dependent variable: overdose death rate	Model 1	Model 2	Model 3	Model 4	Model 5	Model 6
Treatment group	Oregon	Oregon	Washington	Washington	Both states	Both states
Decriminalization period, % (SE) [95% CI]	0.25 (0.07) [0.10 to 0.39]^b^	0.09 (0.11) [−0.11 to 0.31]	0.19 (0.07) [0.04 to 0.34]^b^	−0.12 (0.16) [−0.43 to 0.20]	0.22 (0.07) [0.07 to 0.37]^b^	−0.007 (0.15) [−0.30 to 0.30]
Fentanyl, % (SE) of total seizures [95% CI]	NA	0.02 (0.008) [0.005 to 0.04]^b^	NA	0.02 (0.007) [0.005 to 0.04]^b^	NA	0.02 (0.007) [0.005 to 0.03]^b^
No. of observations	2400	2400	2400	2400	2448	2448

Abbreviation: NA, not applicable.

^a^ Data are presented as the results of 6 difference-in-differences regression models estimating the association between drug decriminalization periods and overdose mortality rates with and without adjustment for fentanyl exposure (estimated with the R package fixest). Odd-numbered columns are direct replications of the Table 4 results in Spencer^18^; each even-numbered column augments an original model by including a fentanyl exposure as a covariate. All models include fixed effects for state and month, and SEs are clustered at the state level.

^b^ *P* < .05.

### Supplementary Analysis

As sensitivity checks, we estimated associations between decriminalization and overdose mortality using the following additional panel data methods for counterfactual inference that accommodate time-varying covariates: difference in differences, generalized synthetic control,^30^ and interactive fixed effects.^29,31^ The results were consistent with our primary analyses: for each method, the crude estimate for decriminalization (without incorporating fentanyl) was positive and, depending on the method, statistically significant. However, after adjusting for fentanyl exposure, the association between decriminalization and overdose mortality was always indistinguishable from 0 (eTable 4 in Supplement 1). In eFigure 4 in Supplement 1, we also found that recriminalization of drug possession as a misdemeanor offense in Washington in late July 2021 was not associated with reductions in overdose death in that state. Rather, overdose mortality rates accelerated in the months after decriminalization was repealed (eFigure 4 in Supplement 1).

## Discussion

To our knowledge, this study is the first to consider the presence of fentanyl in the illicit drug market in Oregon as a time-varying confounder when assessing the consequences of M110 on fatal overdose. Our findings suggest that the increase in the state’s fatal drug overdose rate after implementation of M110 should not be attributed to drug decriminalization, and the state’s contemporaneous transition to a fentanyl-based unregulated drug market is the more plausible explanation. We also observed that the contemporaneous recriminalization of drug possession in Washington coincided with increased drug overdose deaths. These findings are consistent with a 2021 qualitative study in which people who used drugs in Oregon reported that fentanyl had recently dominated the state’s unregulated opioid supply, increasing their risk of overdose.^32^

Despite hopes that the implementation of M110 would lead to a decrease in overdose mortality, deaths increased substantially. It is worth noting that M110 had 2 components: (1) decriminalization and (2) substantial expansion of substance use disorder treatment, recovery, housing, and harm reduction services. Because the majority of the funds to expand these services were not disbursed by the Oregon Health Authority until after August 2022^11^ (18 months after the law took effect), analyses that rely primarily on data between February 1, 2021, and August 31, 2022, only assess the decriminalization component of M110. Moreover, more than 50 years of drug criminalization has likely had persistent effects on behaviors relevant to overdose mortality, such as hesitation to call 911 to report overdose events,^33,34,35^ and acknowledging a highly stigmatized criminal behavior when seeking treatment.^36,37^ We do not know how long it would take for these attitudes and behaviors to change. Regardless, our analysis of Washington State suggests that recriminalization in Oregon may not reduce the rate of overdoses observed in a state saturated with fentanyl.

Analyses of interventions intended to reduce overdose mortality or the consequences of criminalization, ranging from increased naloxone distribution^38^ to relaxed state marijuana laws,^39^ have similarly relied on models that did not account for the spread of fentanyl across the US. Such studies potentially misattribute the consequences of increased fentanyl exposure to the effects of public policies, or they conversely misattribute success to policies because control groups experienced fentanyl supply shocks during the study period. Synthetic control methods and similar causal inference models rely on assumptions of strict exogeneity that are violated when unincorporated time-varying factors (eg, fentanyl spread) are correlated with an exposure (eg, policy adoption) and an outcome of interest (eg, overdose rates).^40,41^ Because shocks to the drug supply drive changes in overdose and affect regions at different times,^21,26^ neglecting this dynamic is a threat to causal inference.

### Limitations

This study has some limitations. There is no way to randomly assign states to an experimental regime of drug decriminalization, so causal relationships between decriminalization and other variables remain uncertain. It is therefore important to consider whether decriminalization itself led to an increase in the supply of unregulated fentanyl by altering supply or demand-side behavioral incentives, in turn leading to increased overdose mortality. Figure 1, however, shows that the 4 states bordering Oregon (Washington, Idaho, Nevada, and California) experienced a surge in fentanyl case reports to the NFLIS during the same period as Oregon, and all later than most other US states. These data suggest that the introduction of fentanyl into the unregulated drug supply would have happened regardless of M110.

Our model did not utilize restricted CDC mortality data, resulting in a small number of cases in which death counts were suppressed to guard against reidentification. However, there is no reason to believe this undercounting was differential based on exposure, confounding, or outcome variables. As with the studies of Spencer^18^ and Joshi et al,^19^ our model used data from the other 48 US states under the assumption that they aggregate to a control version of Oregon that is comparable except for decriminalization, and the validity of our model depends on the degree to which this is accurate. Another limitation is that NFLIS data are an imperfect proxy measurement of fentanyl spread through state-level illicit drug supplies. States vary in the volume of samples reported to the NFLIS (eFigure 5 in Supplement 1). By calculating the share of a state’s NFLIS seizures that contain fentanyl, we adjusted for these absolute reporting differences between states, under the assumption that these reported shares reflect the composition of the underlying market with sufficient accuracy. Public NFLIS data are provided at 6-month intervals, which provides less temporal granularity than mortality data. It also does not fully capture the underlying concept of supply risk in the drug environment, both because law enforcement drug seizures may not always accurately represent the actual drug supply and because other drug supply factors (eg, the presence of fentanyl in counterfeit pills^42^ or contamination of the nonopioid drug supply^43^) affect the risk environment as well. However, our measure tracked the geographic spread of fentanyl in accordance with prior reporting and exhibited an association with fatal overdose trends.

## Conclusions

This cohort study examined the association between fatal drug overdose and the spread of fentanyl through Oregon’s unregulated drug market. Public policies that reduce or eliminate criminal penalties for people who use drugs remain controversial. Because illicitly manufactured fentanyl is a primary driver of fatal overdose rates in the US and the introduction of fentanyl to the nation’s unregulated drug markets occurred in different regions at different times, efforts to evaluate interventions such as M110 should account for changes in the drug supply of the settings under study. After accounting for the fentanyl shock to Oregon’s unregulated drug supply, we did not find evidence of an association between drug decriminalization and overdose mortality. There remains a pressing need for innovative, evidence-based policies to address the nation’s overdose mortality crisis and rigorous, accurate means to assess their effects.

## Notes

Supplement 1. **eTable 1.** Fentanyl Saturation (Per Capita Metric) and Overdose Rates **eTable 2.** Fentanyl Saturation (Standardized Metric) and Overdose Rates **eTable 3.** Matrix Completion Synthetic Control Method Analyses **eTable 4.** Sensitivity Tests With Alternative Panel Data Models **eFigure 1.** Fentanyl Exposure, by State, Over Time **eFigure 2.** Within-State Associations Between Fentanyl Exposure and Overdose Mortality Rates by Region, 2008 to 2022 **eFigure 3.** Difference-in-Differences Results **eFigure 4.** Trend in Overdose Mortality in Washington State Before, During, and After Decriminalization **eFigure 5.** Fentanyl Seizure Rates by State per Half Year (2008-2022)
eMethods

Supplement 2.
Data Sharing Statement

## References

[1] Provisional drug overdose death counts. National Center for Health Statistics. Accessed June 2, 2024. https://www.cdc.gov/nchs/nvss/vsrr/drug-overdose-data.htm

[2] Ray B, Korzeniewski SJ, Mohler G, . Spatiotemporal analysis exploring the effect of law enforcement drug market disruptions on overdose, Indianapolis, Indiana, 2020-2021. Am J Public Health. 2023;113(7):750-758. doi:10.2105/AJPH.2023.307291 37285563 PMC10262257

[3] Hartung DM, McCracken CM, Nguyen T, Kempany K, Waddell EN. Fatal and nonfatal opioid overdose risk following release from prison: a retrospective cohort study using linked administrative data. J Subst Use Addict Treat. 2023;147:208971. doi:10.1016/j.josat.2023.208971 36821990 PMC10795482

[4] Binswanger IA, Blatchford PJ, Mueller SR, Stern MF. Mortality after prison release: opioid overdose and other causes of death, risk factors, and time trends from 1999 to 2009. Ann Intern Med. 2013;159(9):592-600. doi:10.7326/0003-4819-159-9-201311050-00005 24189594 PMC5242316

[5] Townsend TN, Hamilton LK, Rivera-Aguirre A, . Use of an inverted synthetic control method to estimate effects of recent drug overdose Good Samaritan laws, overall and by Black/White race/ethnicity. Am J Epidemiol. 2022;191(10):1783-1791. doi:10.1093/aje/kwac122 35872589 PMC9989361

[6] Hamilton L, Davis CS, Kravitz-Wirtz N, Ponicki W, Cerdá M. Good Samaritan laws and overdose mortality in the United States in the fentanyl era. Int J Drug Policy. 2021;97:103294. doi:10.1016/j.drugpo.2021.103294 34091394 PMC9529169

[7] *Report of the National Survey to Assess First Responder Deflection Programs in Response to the Opioid Crisis: Final Report*. NORC at the University of Chicago, TASC Center for Health and Justice; 2021. Accessed December 27, 2023. https://www.cossup.org/Content/Documents/Articles/CHJ-TASC_Nation_Survey_Report.pdf

[8] Siddiqui ST, La Manna A, Connors E, . An evaluation of first responders’ intention to refer to post-overdose services following SHIELD training. Harm Reduct J. 2024;21(1):39. doi:10.1186/s12954-024-00957-4 38351046 PMC10863209

[9] Rouhani S, Zhang L, Winiker AK, Sherman SG, Bandara S. Emerging models of de facto drug policy reforms in the United States. Drug Alcohol Depend. 2024;260:111341. doi:10.1016/j.drugalcdep.2024.111341 38815292

[10] Pozo BD, Krasner LS, George SF. Decriminalization of diverted buprenorphine in Burlington, Vermont and Philadelphia: an intervention to reduce opioid overdose deaths. J Law Med Ethics. 2020;48(2):373-375. doi:10.1177/1073110520935353 32631187 PMC9197600

[11] Russoniello K, Vakharia SP, Netherland J, . Decriminalization of drug possession in Oregon: analysis and early lessons. Drug Sci Policy Law. 2023;9:1-16. doi:10.1177/20503245231167407

[12] Davis CS, Joshi S, Rivera BD, Cerdá M. Changes in arrests following decriminalization of low-level drug possession in Oregon and Washington. Int J Drug Policy. 2023;119:104155. doi:10.1016/j.drugpo.2023.104155 37567089

[13] Good D, Leichtling G, Pustejovsky S. *Oregon Decriminalizes Drugs: A State-Level Process Evaluation of Early Implementation*. Comagine Health and Vital Strategies; 2023. Accessed May 27, 2024. https://comagine.org/sites/default/files/resources/oregon-decriminalizes-drugs.pdf

[14] Crombie N. Drug treatment providers slow to spend Measure 110 dollars, some counties serving few people, new audit finds. *The Oregonian*. December 20, 2023. Accessed May 28, 2024. https://www.oregonlive.com/news/2023/12/treatment-providers-slow-to-spend-measure-110-dollars-some-counties-serving-few-people-new-audit-finds.html

[15] Smiley-McDonald HM, Attaway PR, Wenger LD, Greenwell K, Lambdin BH, Kral AH. “All carrots and no stick”: perceived impacts, changes in practices, and attitudes among law enforcement following drug decriminalization in Oregon State, USA. Int J Drug Policy. 2023;118:104100. doi:10.1016/j.drugpo.2023.104100 37356287

[16] Terry L. Latest data show overdoses continue to skyrocket in Oregon. Oregon Public Broadcasting. Updated January 28, 2024. Accessed July 1, 2024. https://www.opb.org/article/2024/01/28/data-show-overdoses-deaths-rising-in-oregon/

[17] Baker M. Oregon is recriminalizing drugs. *New York Times*. April 1, 2024. Accessed May 3, 2024. https://www.nytimes.com/2024/04/01/us/oregon-drug-law-portland-mayor.html

[18] Spencer N. Does drug decriminalization increase unintentional drug overdose deaths? early evidence from Oregon Measure 110. J Health Econ. 2023;91:102798. doi:10.1016/j.jhealeco.2023.102798 37556870

[19] Joshi S, Rivera BD, Cerdá M, . One-year association of drug possession law change with fatal drug overdose in Oregon and Washington. JAMA Psychiatry. 2023;80(12):1277-1283. doi:10.1001/jamapsychiatry.2023.3416 37755815 PMC10535015

[20] Ciccarone D. The triple wave epidemic: supply and demand drivers of the US opioid overdose crisis. Int J Drug Policy. 2019;71:183-188. doi:10.1016/j.drugpo.2019.01.010 30718120 PMC6675668

[21] Zoorob M. Fentanyl shock: the changing geography of overdose in the United States. Int J Drug Policy. 2019;70:40-46. doi:10.1016/j.drugpo.2019.04.010 31079029

[22] Zibbell JE, Aldridge AP, Cauchon D, DeFiore-Hyrmer J, Conway KP. Association of law enforcement seizures of heroin, fentanyl, and carfentanil with opioid overdose deaths in Ohio, 2014-2017. JAMA Netw Open. 2019;2(11):e1914666. doi:10.1001/jamanetworkopen.2019.14666 31702795 PMC6902770

[23] Ciccarone D. Fentanyl in the US heroin supply: a rapidly changing risk environment. Int J Drug Policy. 2017;46:107-111. doi:10.1016/j.drugpo.2017.06.010 28735776 PMC5742018

[24] Drug overdose deaths in the United States, 2001–2021. *NCHS Data Brief*. Centers for Disease Control and Prevention. December 2022. Accessed December 27, 2023. https://www.cdc.gov/nchs/data/databriefs/db457.pdf

[25] Mars SG, Rosenblum D, Ciccarone D. Illicit fentanyls in the opioid street market: desired or imposed? Addiction. 2019;114(5):774-780. doi:10.1111/add.14474 30512204 PMC6548693

[26] Shover CL, Falasinnu TO, Dwyer CL, . Steep increases in fentanyl-related mortality west of the Mississippi River: recent evidence from county and state surveillance. Drug Alcohol Depend. 2020;216:108314-108314. doi:10.1016/j.drugalcdep.2020.108314 33038637 PMC7521591

[27] Athey S, Bayati M, Doudchenko N, Imbens G, Khosravi K. Matrix completion methods for causal panel data models. J Am Stat Assoc. 2021;116(536):1716-1730. doi:10.1080/01621459.2021.1891924

[28] Killick R, Eckley IA. changepoint: An R package for changepoint analysis. J Stat Softw. 2014;58:1-19. doi:10.18637/jss.v058.i03

[29] Liu L, Wang Y, Xu Y. A practical guide to counterfactual estimators for causal inference with time-series cross-sectional data. Am J Pol Sci. 2024;68(1):160-176. doi:10.1111/ajps.12723

[30] Xu Y. Generalized synthetic control method: causal inference with interactive fixed effects models. Polit Anal. 2017;25(1):57-76. doi:10.1017/pan.2016.2

[31] Gobillon L, Magnac T. Regional policy evaluation: interactive fixed effects and synthetic controls. Rev Econ Stat. 2016;98(3):535-551. doi:10.1162/REST_a_00537

[32] Shin SS, LaForge K, Stack E, . “It wasn’t here, and now it is. It’s everywhere”: fentanyl’s rising presence in Oregon’s drug supply. Harm Reduct J. 2022;19(1):76. doi:10.1186/s12954-022-00659-9 35818072 PMC9275036

[33] Pozo BD. Reducing the iatrogenesis of police overdose response: time is of the essence. Am J Public Health. 2022;112(9):1236-1238. doi:10.2105/AJPH.2022.306987 35862886 PMC9382163

[34] Pamplin JR II, Rouhani S, Davis CS, King C, Townsend TN. Persistent criminalization and structural racism in US drug policy: the case of overdose Good Samaritan laws. Am J Public Health. 2023;113(S1):S43-S48. doi:10.2105/AJPH.2022.307037 36696623 PMC9877371

[35] Atkins DN, Del Pozo B, Clark MH, Andraka-Christou B, O’Donnell D, Ray B. Disparities in the accuracy of reporting opioid overdoses to 9-1-1 by race and sex of overdose victim, Marion County, Indiana, 2011-2020. Health Justice. 2024;12(1):25. doi:10.1186/s40352-024-00279-4 38819492 PMC11143637

[36] Scher BD, Neufeld SD, Butler A, . “Criminalization causes the stigma”: perspectives from people who use drugs. Contemp Drug Probl. 2023;50(3):402-425. doi:10.1177/00914509231179226

[37] Newman BN, Crowell KA. The intersectionality of criminality and substance use self-stigmas. Stigma Health. 2023;8(2):212-222. doi:10.1037/sah0000293

[38] Doleac JL, Mukherjee A. The effects of naloxone access laws on opioid abuse, mortality, and crime. J Law Econ. 2022;65(2):211-238. doi:10.1086/719588

[39] Mathur NK, Ruhm CJ. Marijuana legalization and opioid deaths. J Health Econ. 2023;88:102728. doi:10.1016/j.jhealeco.2023.102728 36808015

[40] Hollingsworth A, Wing C. Tactics for design and inference in synthetic control studies: an applied example using high-dimensional data. SSRN. 2020:3592088. doi:10.2139/ssrn.3592088

[41] Powell D. Synthetic control estimation beyond comparative case studies: does the minimum wage reduce employment? J Bus Econ Stat. 2022;40(3):1302-1314. doi:10.1080/07350015.2021.1927743

[42] O’Donnell J, Tanz LJ, Miller KD, . Drug overdose deaths with evidence of counterfeit pill use—United States, July 2019–December 2021. MMWR Morb Mortal Wkly Rep. 2023;72(35):949-956. doi:10.15585/mmwr.mm7235a3 37651284

[43] Park JN, Rashidi E, Foti K, Zoorob M, Sherman S, Alexander GC. Fentanyl and fentanyl analogs in the illicit stimulant supply: results from U.S. drug seizure data, 2011-2016. Drug Alcohol Depend. 2021;218:108416. doi:10.1016/j.drugalcdep.2020.108416 33278761 PMC7751390

