# Errors in Figures

**DOI:** 10.1001/jamanetworkopen.2024.43755

**Published:** 2024-10-16

In the Original Investigation titled “Drug Decriminalization, Fentanyl, and Fatal Overdoses in Oregon,”^1^ published September 5, 2024, the y-axis labels in Figures 2 and 3 erroneously included a percent symbol (%) at the end of the label. This article has been corrected.
