# Supplementary material for: Drug Decriminalization, Fentanyl, and Fatal Overdoses in Oregon
Source: JAMA Netw Open. 2024 Sep 5;7(9):e2431612. doi: 10.1001/jamanetworkopen.2024.31612 (PMC11378001; doi:10.1001/jamanetworkopen.2024.31612)
Supplement: Supplement 1. — eTable 1. Fentanyl Saturation (Per Capita Metric) and Overdose Rates eTable 2. Fentanyl Saturation (Standardized Metric) and Overdose Rates eTable 3. Matrix Completion Synthetic Control Method Analyses eTable 4. Sensitivity Tests With Alternative Panel Data Models eFigure 1. Fentanyl Exposure, by State, Over Time eFigure 2. Within-State Associations Between Fentanyl Exposure and Overdose Mortality Rates by Region, 2008 to 2022 eFigure 3. Difference-in-Differences Results eFigure 4. Trend in Overdose Mortality in Washington State Before, During, and After Decriminalization eFigure 5. Fentanyl Seizure Rates by State per Half Year (2008-2022) eMethods [file jamanetwopen-e2431612-s001.pdf]

Supplementary Online Content

Zoorob MJ, Park JN, Kral AH, Lambdin BH, del Pozo B. Drug decriminalization, fentanyl, and fatal overdoses in Oregon. *JAMA Netw Open*. 2024;7(9):e2431612. doi:10.1001/jamanetworkopen.2024.31612

eTable 1. Fentanyl Saturation (Per Capita Metric) and Overdose Rates

eTable 2. Fentanyl Saturation (Standardized Metric) and Overdose Rates

eTable 3. Matrix Completion Synthetic Control Method Analyses

eTable 4. Sensitivity Tests With Alternative Panel Data Models

eFigure 1. Fentanyl Exposure, by State, Over Time

eFigure 2. Within-State Associations Between Fentanyl Exposure and Overdose Mortality Rates by Region, 2008 to 2022

eFigure 3. Difference-in-Differences Results

eFigure 4. Trend in Overdose Mortality in Washington State Before, During, and After Decriminalization

eFigure 5. Fentanyl Seizure Rates by State per Half Year (2008-2022)

eMethods

This supplementary material has been provided by the authors to give readers additional information about their work.

© 2024 Zoorob MJ et al. *JAMA Network Open.*

eTable 1. Fentanyl Saturation (Per Capita Metric) and Overdose Rates

**Dep Var: Overdose Death Rate**	(1)	(2)	(3)	(4)	(5)
Fentanyl seizures per capita (*asinh*)	2.53* (0.06) [2.41, 2.65]	2.48* (0.15) [2.17, 2.79]	2.30* (0.10) [2.10, 2.50]	1.86* (0.22) [1.41, 2.30]	1.10* (0.25) [0.60, 1.59]
State Fixed effects	No	Yes	No	Yes	Yes
Time (Half-Year) Fixed Effects	No	No	Yes	Yes	Yes
State Linear Time Trend	No	No	No	No	Yes
S.E. Clustering	None	State	Half Year	State	State
Observations	1,530	1,530	1,530	1,530	1,530

Table shows coefficients, standard errors (SE), and confidence intervals (CI) of fentanyl exposure (operationalized as the inverse hyperbolic sine transform or asinh of fentanyl seizures per 100,000 people) on overdose death mortality rates. The unit of analysis is state-half (e.g., Alabama in H1 of 2010). Date range is 2008 H1 (January 2008 to June 2008) to 2022 H2 (July 2022 to December 2022). Standard errors in parentheses. * $p < 0.05$

© 2024 Zoorob MJ et al. *JAMA Network Open.*

eTable 2. Fentanyl Saturation (Standardized Metric) and Overdose Rates

**Dep Var: Overdose Death Rate**	(1)	(2)	(3)	(4)	(5)
Fentanyl saturation index	2.24* (0.06)<br>[2.13, 2.34]	2.29* (0.18)<br>[1.93, 2.65]	1.85* (0.08)<br>[1.68, 2.01]	1.68* (0.24)<br>[1.19, 2.17]	1.18* (0.23)<br>[0.71, 1.65]
State Fixed effects	No	Yes	No	Yes	Yes
Time (Half-Year) Fixed Effects	No	No	Yes	Yes	Yes
State Linear Time Trend	No	No	No	No	Yes
S.E. Clustering	None	State	Half Year	State	State
Observations	1,530	1,530	1,530	1,530	1,530

Table shows coefficients, standard errors (SE), and confidence intervals (CI) of fentanyl exposure (operationalized as a standardized index of two components: first, the percent of NFLIS records that were for what the laboratory defined as "fentanyl or related substances" and, second, the inverse hyperbolic sine transform of fentanyl seizures per capita) on overdose death mortality rates. Unit of analysis is state-half (e.g., Alabama in H1 of 2010). Date range is 2008 H1 to 2022 H2. Standard errors in parentheses. * implies $p < 0.05$. Estimates were conducted using the *fixest* package in R.

© 2024 Zoorob MJ et al. *JAMA Network Open.*

eTable 3. Matrix Completion Synthetic Control Method Analyses

	Model 1	Model 2
Coefficient	Estimate (SE) [95% CI lower, upper]	Estimate (SE) [95% CI lower, upper]
Decriminalization	1.83* (0.47) [0.92, 2.74]	-0.51 (0.61) [-1.70, 0.69]
Fentanyl		0.16* (0.03) [0.09, 0.22]
Observations	1500	1500
RMSE	0.47	0.45

* $p < 0.05$. Jackknife standard errors in parentheses and confidence intervals in brackets. The Root Mean Square Error (RMSE), a measure of model fit, is shown to highlight the improved model fit in the model controlling for fentanyl. Estimates were conducted using the *fect* package in R.

© 2024 Zoorob MJ et al. *JAMA Network Open.*

eTable 4. Sensitivity Tests With Alternative Panel Data Models

Dep Var: Overdose Death Rate	DID 1	DID 2	IFE 1	IFE 2	Gsynth 1	Gsynth 2
Decriminalization	0.35 (0.70) [-1.04, 1.75]	-0.88 (0.69) [-2.26, 0.50]	1.43 (0.88) [-0.30, 3.17]	-0.79 (0.70) [-2.15, 0.58]	2.50 (0.45) [1.62, 3.39]	1.48 (1.44) [-1.44, 4.29]
Fentanyl Exposure		0.16* (0.03) [0.10, 0.22]		0.16* (0.03) [0.09, 0.22]		0.12* (0.02) [0.08, 0.16]
Standard Errors	Parametric, clustered by state	Parametric, clustered by state	Jackknife	Jackknife	Jackknife	Jackknife
N	1500	1500	1500	1500	1500	1500

Standard errors in parenthesis. 95% confidence intervals in brackets. * p < 0.05

This table shows crude and adjusted results of the association of decriminalization on overdose mortality with three alternative panel data methods. The first pair of columns shows results from difference-in-differences (DID) two way fixed effect regressions; the second pair of columns shows results from the Interactive Fixed Effects (IFE) method, and the third pair shows results from the generalized synthetic control method (Gsynth). Following the approach we present in the main analyses, for each method, an unadjusted model including only an indicator for decriminalization is shown first, followed by a model that adjusts for fentanyl exposure. Estimates were conducted using the *fect* package in R.

© 2024 Zoorob MJ et al. *JAMA Network Open.*

eFigure 1. Fentanyl Exposure, by State, Over Time

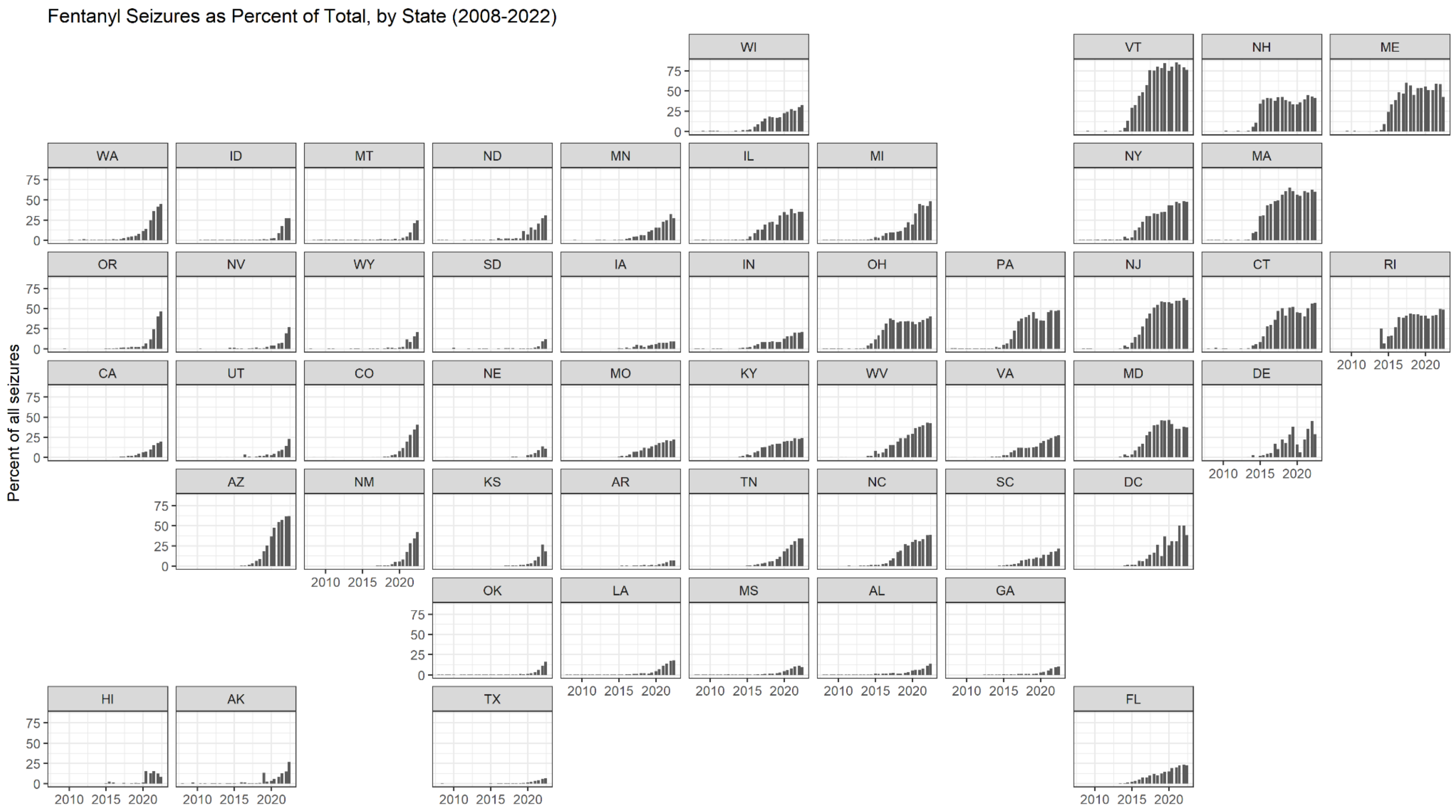

States are arranged by their approximate geographic location. The x-axis is the date in half-years from H1 2008 to H2 2022. The y-axis is the percent of all NFLIS seizures that contain a substance that NFLIS categorizes as “fentanyl or a related substance.

© 2024 Zoorob MJ et al. *JAMA Network Open.*

eFigure 2. Within-State Associations Between Fentanyl Exposure and Overdose Mortality Rates by Region, 2008 to 2022

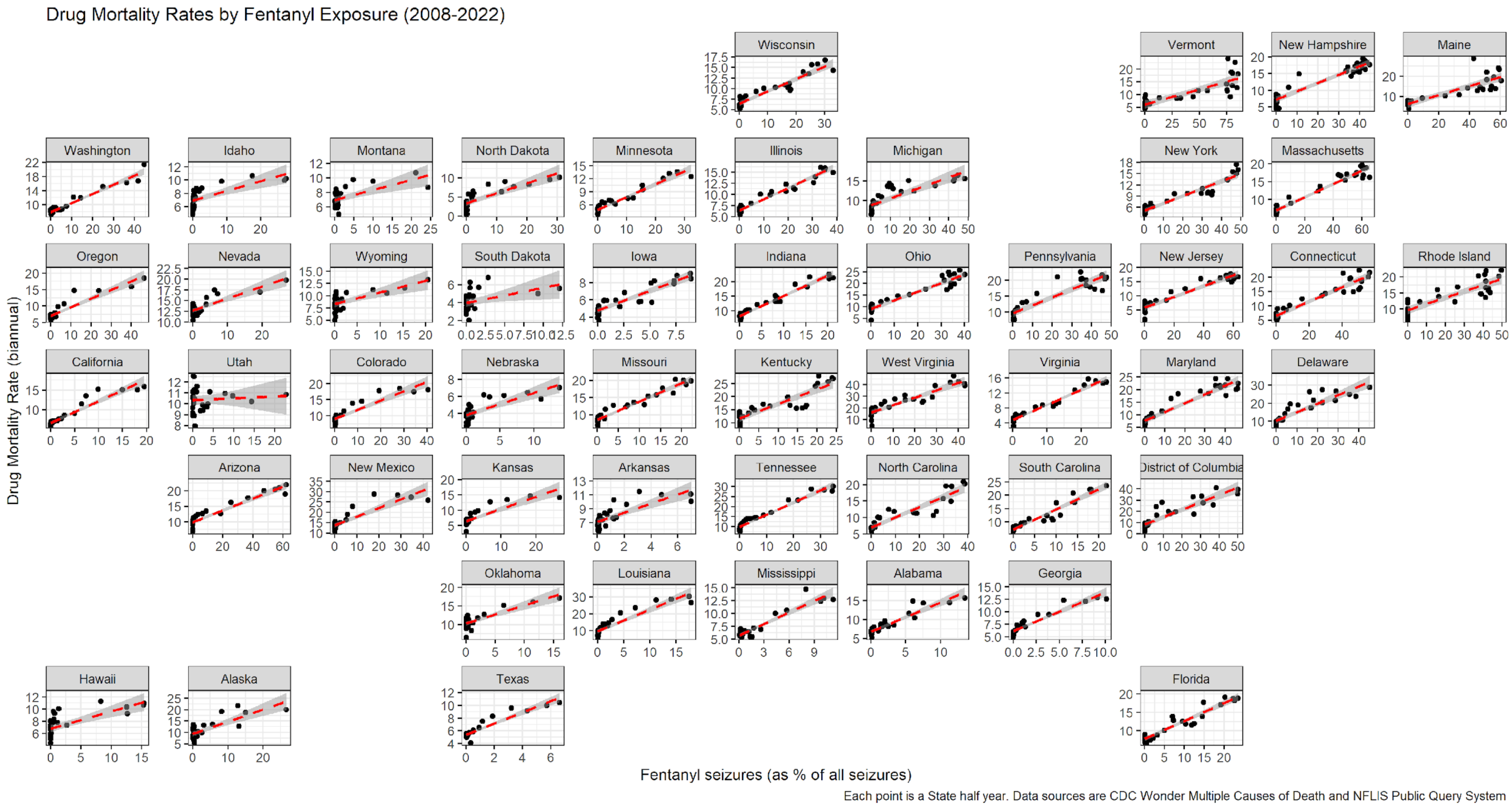

This Figure illustrates the relationship between the percent of all NFLIS law enforcement seizures that contain fentanyl and drug overdose mortality rates, with states roughly arranged geographically (via the *geofacet* R package). Each point represents a half-year period in a state over the period 2008-2022. In virtually all states, a strong positive relationship exists between fentanyl exposure and overdose mortality

© 2024 Zoorob MJ et al. *JAMA Network Open.*

eFigure 3. Difference-in-Differences Results

Effect of drug decriminalization on overdose mortality unadjusted, and adjusted for the rapid escalation of fentanyl by state

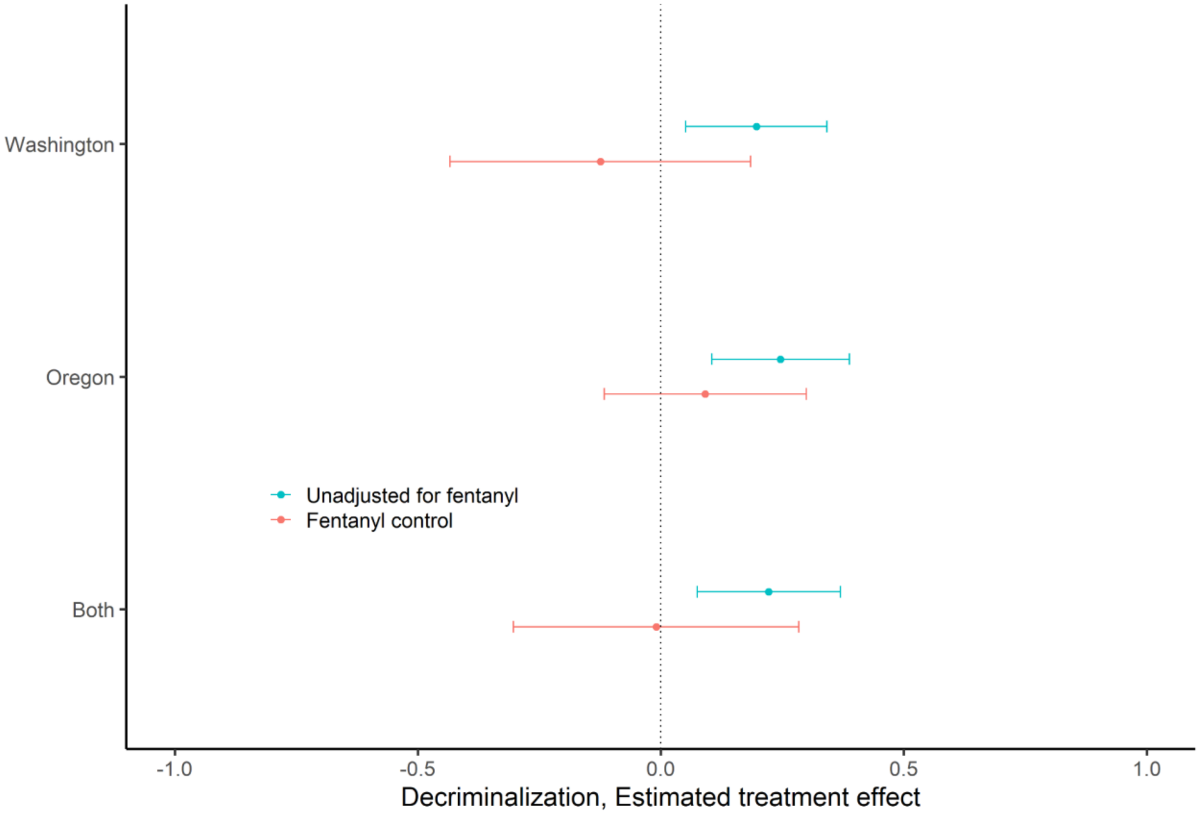

This plot highlights the impact of adjusting for fentanyl on the estimated effect of decriminalization on overdose mortality. Blue point estimates and 95% confidence intervals replicate main effect difference-in-difference results in Table 1 of Spencer 2023. Red point estimates and confidence intervals show the estimated treatment effect of decriminalization adjusting for the percent of fentanyl in NFLIS seizures.

© 2024 Zoorob MJ et al. *JAMA Network Open.*

eFigure 4. Trend in Overdose Mortality in Washington State Before, During, and After Decriminalization

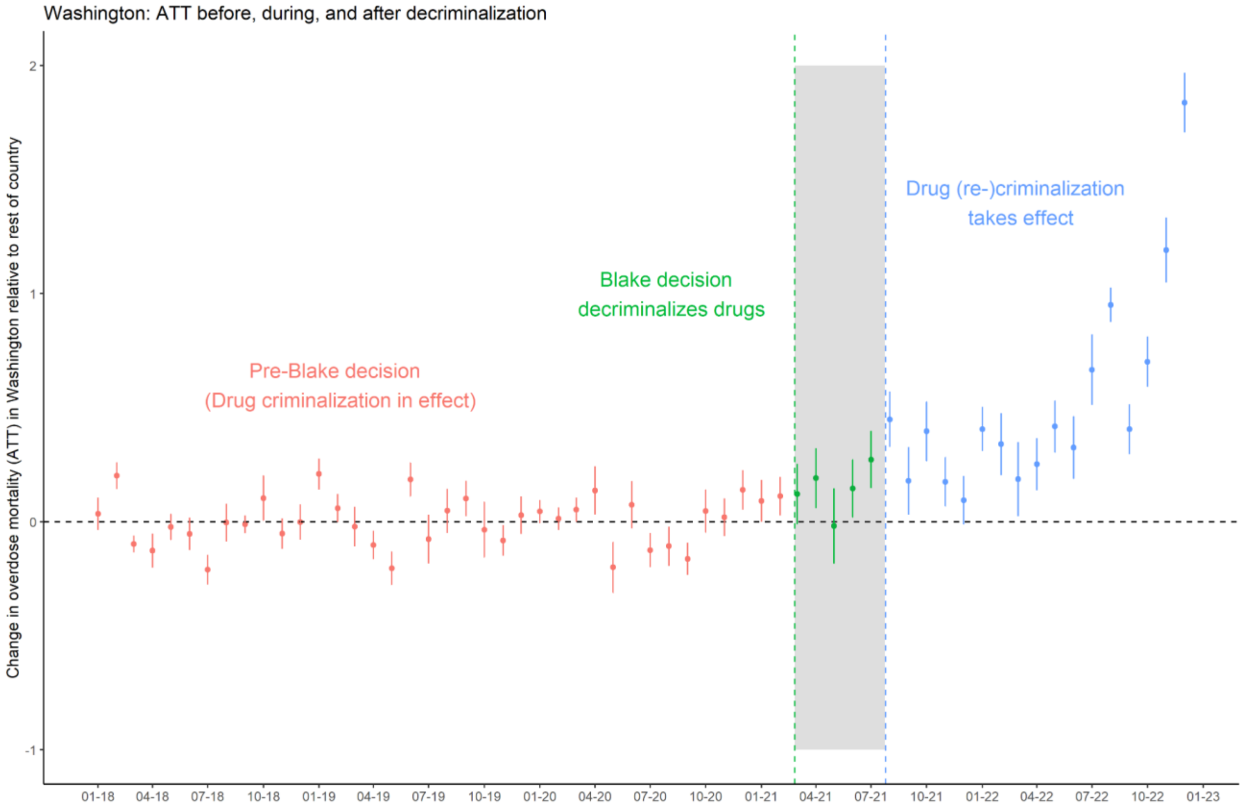

This plot shows the trend in overdose mortality in Washington (relative to a synthetic control estimated through the Matrix Completion method without covariates) in the periods before the Blake decision decriminalized drugs (from January 2018 through February 2021; shaded red), during the 5-months of decriminalization (March 2021 through July 2021; shaded green), and in the months after drugs were decriminalized (August 2021 through December 2022; shaded blue). Each point represents the Average Treatment Effect on the Treated (ATT) for Washington in each month, with 95% confidence intervals.

© 2024 Zoorob MJ et al. *JAMA Network Open.*

eFigure 5. Fentanyl Seizure Rates by State per Half Year (2008-2022)

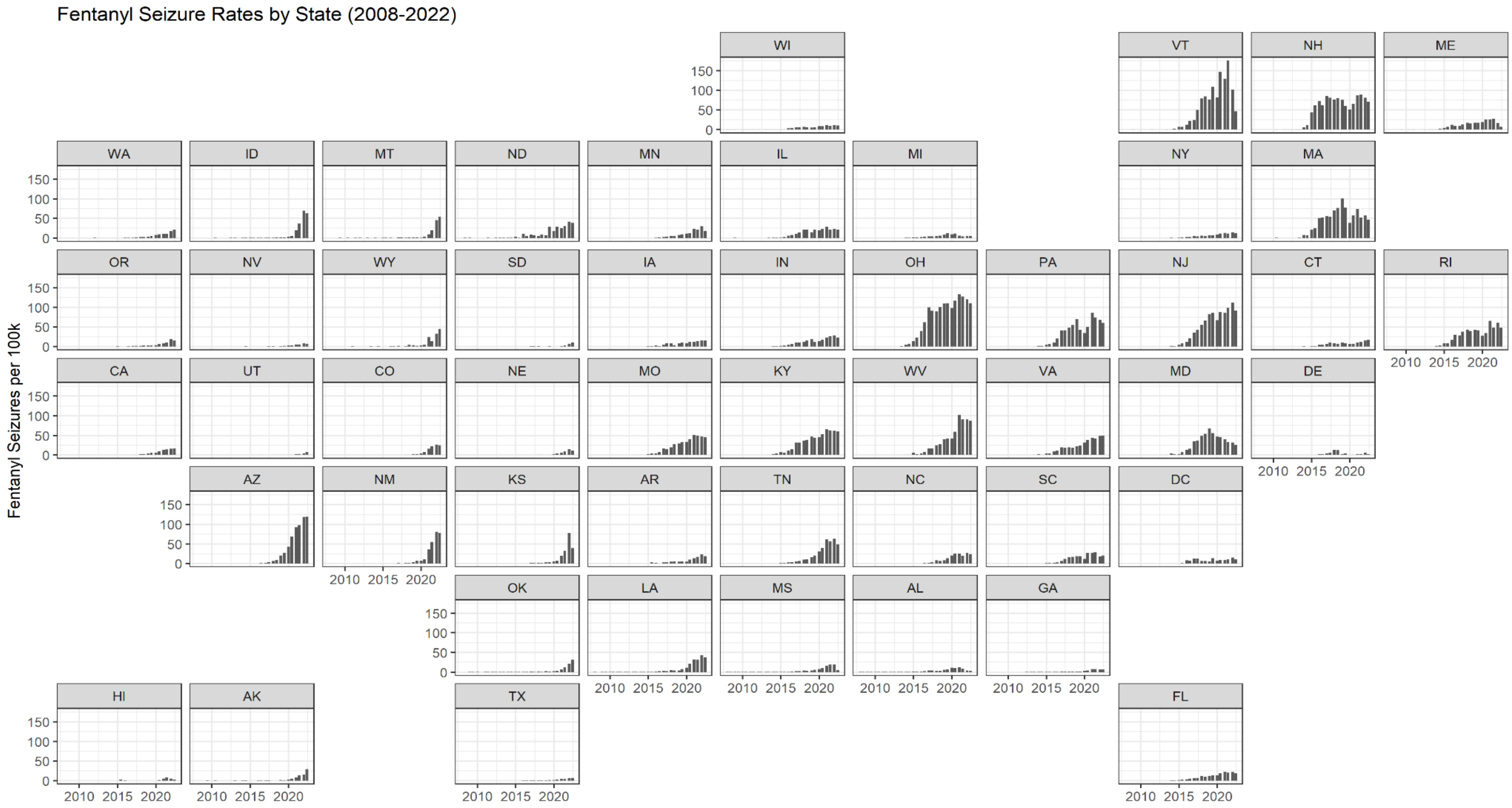

This figure shows the biannual count of samples containing fentanyl or a related substance (per 100,000 residents) in the NFLIS data, by state. The plot highlights the relative differences between states in the magnitude of samples, with some states (adjusting for population) contributing substantially more samples to NFLIS. This heterogeneity indicates the advantage of using the share of samples that contain fentanyl within each state (eFigure1), which adjusts for heterogeneity between states in their reporting practices to NFLIS.

© 2024 Zoorob MJ et al. *JAMA Network Open.*

eMethods

Additional details

Notes on Mortality Data

Specifically, we used the mortality codes Multiple Causes of Death (MCD) Drug/Alcohol Induced Causes: Drug poisonings (overdose) Unintentional (X40-X44); Drug poisonings (overdose) Suicide (X60-X64); Drug poisonings (overdose) Homicide (X85); Drug poisonings (overdose) Undetermined (Y10-Y14). The 2022 death statistics were described as provisional. In replications of Spencer 2023, we used the following codes: Underlying Cause of Death (UCD) - Drug/Alcohol Induced Causes: Drug poisonings (overdose) Unintentional (X40-X44)

Re-criminalization in Washington

Both Oregon and Washington decriminalized simple possession of controlled substances in early 2021: in Oregon, M110, approved by the voters in November 2020, took effect February 1st, whereas Washington's state supreme court on February 25, 2021 struck down the statute criminalizing drugs in the decision *State v Blake*. The policy trajectories of the two states differed substantially thereafter, however. In April 2021, in response to the Blake decision, the Washington legislature passed two-year legislation (SB 5476) that restored criminal penalties for simple drug possession with an effective date of July 25, 2021 (the legislature later passed a permanent law criminalizing drug possession that took effect in 2023). Meanwhile, Oregon maintained decriminalization throughout the study period.

If decriminalization, rather than a regional fentanyl saturation, caused the observed increases in overdose deaths in these states, we might expect overdose mortality trends in Washington to deviate from Oregon after Washington re-criminalized drug possession. In Washington, drug decriminalization was in effect from February 25, 2021 to July 25, 2021.

We use the same Matrix Completion method as in the main analysis, but with monthly overdose mortality data, comparing the monthly change in overdose in Washington relative to an imputed counterfactual. These estimates are plotted in eFigure3, with the estimates prior to the decriminalization period shaded red, estimates during the decriminalization period shaded green, and estimates after the repeal of decriminalization shaded blue. As eFigure3 illustrates, trends in overdose deaths in Washington *substantially accelerated* after the state re-criminalized controlled substances. Overdose mortality increased to a much larger degree at the end of 2022 – more than a year after the legislature

© 2024 Zoorob MJ et al. *JAMA Network Open.*

had passed a law making drug possession a misdemeanor – than in the 5 months that drug decriminalization was in effect.

Washington's unique policy trajectory influences our interpretation of results. There may be carryover effects of the *Blake* decision that continue to shape police and consumer behavior after the state re-criminalized drugs. Washington's post-*Blake* law making simple drug possession a misdemeanor offense was unique, and less punitive, than many other states, requiring treatment referrals for the first two offenses.

© 2024 Zoorob MJ et al. *JAMA Network Open.*
