# Supplementary material for: Drug Decriminalization, Fentanyl, and Fatal Overdoses in Oregon
Source: JAMA Netw Open. 2024 Sep 5;7(9):e2431612. doi: 10.1001/jamanetworkopen.2024.31612 (PMC11378001; doi:10.1001/jamanetworkopen.2024.31612)
Supplement: Supplement 2. — Data Sharing Statement [file jamanetwopen-e2431612-s002.pdf]

Data Sharing Statement

Zoorob. Drug Decriminalization, Fentanyl, and Fatal Overdoses in Oregon. *JAMA Netw Open*. Published September 05, 2024. doi:10.1001/jamanetworkopen.2024.31612

Data
Data available: Yes
Data types: Data (not involving human participants)
How to access data: The Centers for Disease Control and Prevention public website
When available: With publication

Supporting Documents
Document types: Statistical/analytic code
How to access documents:https://dataverse.harvard.edu/dataset.xhtml?persistentId=doi:10.7910/DVN/JT4CDS.
When available: With publication

Additional Information
Who can access the data: The data is already in the public domain and may be used by any person for any purpose.
Types of analyses: The data is already in the public domain and may be used by any person for any purpose.
Mechanisms of data availability: Without investigator support.
Any additional restrictions: None.
